# Influence of Shift Work on The Health of Nursing Professionals

**DOI:** 10.3390/jpm13040627

**Published:** 2023-04-02

**Authors:** Pablo Roman, Isabel Perez-Cayuela, Esther Gil-Hernández, Miguel Rodriguez-Arrastia, Adrian Aparicio-Mota, Carmen Ropero-Padilla, Lola Rueda-Ruzafa

**Affiliations:** 1 Faculty of Health Sciences, Department of Nursing, Physiotherapy and Medicine, University of Almeria, 04120 Almeria, Spain; 2 Health Research Center CEINSA, University of Almería, 04120 Almeria, Spain; 3 Torrecardenas University Hospital, 04009 Almeria, Spain; 4 Andalusian Public Foundation for Biomedical Research in Eastern Andalusia (FIBAO), University Hospital Torrecárdenas, 04009 Almeria, Spain

**Keywords:** shift work, nursing, stress, anxiety, sleep quality, work environment

## Abstract

Shift work is increasingly common in health services, subjecting healthcare professionals to work schedules that can alter circadian rhythms and eating habits with consequent repercussions for the intestinal homeostasis. The objective of this study was to describe the relationship of rotating work shifts with the intestinal health, sleep quality, and emotional dimension of nursing professionals. In March and May 2019, an observational and comparative study was conducted among 380 nursing professionals from different Spanish cities and divided into fixed shift (*n* = 159) and rotating shift (*n* = 221). To carry out the present work, the variables measured were gastrointestinal symptoms, stool consistency and shape, anxiety, depression, sleep, as well as stress and work environment. Nurses with rotating shifts reported more abdominal pain and symptoms of depersonalization, as well as worse sleep efficiency and worse nursing practice environment. In addition, overall scores of the Gastrointestinal Symptom Rating Scale and Hospital Anxiety and Depression Scale were found to be significantly worse in nurses with these shifts. Rotating shift work by nursing staff may be associated with the occurrence of gastrointestinal and anxiety-related symptoms. These findings, together with the presence of negative and insensitive attitudes towards patients by nurses on rotating shifts, should be considered to maintain the quality of healthcare.

## 1. Introduction

Shift work in the healthcare sector is often irregularly distributed to ensure round-the-clock patient care. Work schedules generally include fixed and rotating shifts, both of which have been shown to affect the health and quality of life of healthcare workers due to circadian misalignment [1]. In this vein, the consequences for the health and well-being of nurses working irregular and rotating shifts are diverse and include gastrointestinal disorders due to changes in eating habits, sleep disturbances, stress, job strain, personal dissatisfaction at work, and deterioration of social and family relationships [2,3,4].

Access to regular, nutritious meals is a basic human need. However, the reality is that certain working conditions, such as rotating shift work, can severely impact workers’ abilities to meet these fundamental needs. Workers in these circumstances face numerous challenges, including disruptions to their eating patterns, limited access to warm meals, and insufficient caloric intake. This can have serious consequences for their health and well-being, both in the short and long term [5]. Studies have shown that shift and night workers, some of whom have a history of digestive disorders, have more complaints or aggravations of their gastrointestinal symptoms, mainly gastro-esophageal reflux (GER), than day workers [6,7]. These disorders are thought to be related to circadian cycles, as gastrointestinal function differs according to the time of day. With respect to GER, it is known that the environment in the stomach becomes more acidic at night [8,9].

Shift workers experience an imbalance in sleep–wake cycles which can result in fatigue with consequent sleep distortion, as daytime sleep does not have the restorative characteristics of nighttime sleep [10,11]. Different studies show that shift work generates a worsening of sleep quality when compared to daytime work in individuals with different occupations [12,13]. In this regard, abnormalities in sleep pattern lead to drowsiness and can, therefore, affect cognitive function, alertness, and work performance [14,15,16]. All of these disturbances result in an increase in workplace mistakes and in risk of accidents, distractions, and injuries [10,17].

The disruption of the circadian clock system also has an impact on mental health in shift workers by interfering with the hypothalamic–pituitary–adrenal (HPA) axis [18,19]. Cortisol, the HPA axis hormone, which is essential in stressor responses, is affected in night shifts and rotating shifts. Cortisol levels are highest minutes after waking and decrease sharply throughout the day, reaching their lowest point during sleep [19,20]. On this basis, nurses on night shifts sleep during the day and are active at night, thus providing care to patients under stressful conditions. Therefore, cortisol levels remain elevated, which would explain the increase in cortisol secretion with physical or mental stress [20]. In addition, both psychological and physiological stress, consequences of shift work, are some of the most influential factors in the development of digestive disorders, leading to alterations in the composition and function of the gut microbiota and increased intestinal permeability [21].

Situations of chronic stress due to long working hours, inadequate sleep, less physical activity, and shift work can lead to so-called burnout syndrome, characterized by emotional exhaustion, depersonalization, or lack of personal fulfilment [22]. It has been reported that the incidence of burnout has increased among healthcare workers, particularly when compared to the general working population [23]. Experiencing burnout is considered a major factor in nurses leaving or considering leaving their jobs, mainly due to the stressful work environment and lack of staff [24].

In brief, there are many harmful effects of shift work or night work since the circadian rhythm is not adjusted to the normal cycle. Various negative effects include disturbances to the digestive system, sleep and mood disorders, and impairment of cognitive performance [6,19,25]. This study aims to describe effects of shift work on gastrointestinal and digestive complaints, the quality of sleep, and the emotions of nursing staff.

## 2. Materials and Methods

### 2.1. Design

A comparative cross-sectional study was conducted in the first semester of 2019, following the Strengthening the Reporting of Observational Studies in Epidemiology (STROBE) recommendations [26].

### 2.2. Participants

Convenience sampling was used to select our participants. In addition, snowball sampling was used to increase the sample size. Four hundred nursing professionals were recruited. After calculating the sample size, with a confidence interval of 90% and an error margin of 5%, the number of patients to be included was 273 [27]. Finally, the sample consisted of 380 nursing professionals from different Spanish cities. The selection criteria for participants are shown in Table 1. Data collection was designed to ensure confidentiality and anonymity, and participants gave informed consent prior to the study, with the possibility of withdrawing at any time.

### 2.3. Data Collection

Data were collected using validated questionnaires in Spanish to investigate stool consistency and shape, anxiety and depression, sleep, and finally, work environment and burnout syndrome.

Digestive symptomatology was assessed using the Gastrointestinal Symptom Rating Scale (GSRS) [28]. This scale included five subscales: reflux, diarrhea, constipation, abdominal pain, and indigestion. It has a score based on a 7-grade Likert-type scale, where 1 represents the most positive option and 7 the most negative.

Stool consistency and shape were assessed using the Bristol scale [29,30]. This scale consists of 7 items in which 7 stool types are visually and descriptively described. Stool types 1 and 2 are considered abnormally hard stools related to constipation, types 3–5 are generally considered normal stools, and types 6 and 7 are abnormally liquid stools related to diarrhea [31].

Job stress, or Burnout Syndrome, was assessed using the Maslach Burnout Inventory-Human Services Survey (MBI-HSS) questionnaire [32,33]. It consists of 3 scales with 22 items measuring the frequency with which professionals perceive low self-fulfillment at work (8 items), emotional exhaustion (9 items), and depersonalization (5 items). High scores on the emotional exhaustion and depersonalization subscales (27–54 and 10–30, respectively) and low scores on the personal accomplishment subscale (0–33), the third subscale, define Burnout Syndrome.

At an emotional level, anxiety and depression were assessed using the Hospital Anxiety and Depression Scale (HADS) [34,35]. This scale consists of 14 items, of which 7 measure anxiety and 7 measure depression. Each item is scored on a scale with 4 alternatives ranging between 0 and 3 and a total score ranging between 0 and 21, with anxiety and depression assessed separately.

The work environment was measured using the Practice Environment Scale from Revised Nurse Work Index (PES-NWI) [36,37], which consisted of 31 items. It is considered one of the best instruments for measuring the nursing practice environment. Its structure is divided into 5 subscales: (1) nurse involvement in hospital affairs (8 items); (2) nursing rationale for quality of care (9 items); (3) nurse manager, leadership, and support for nurses (4 items); (4) staffing and resources (4 items); and (5) collegial nurse–physician relationships (7 items). It was measured using a 4-point Likert scale ranging from 1 (strongly disagree) to 4 (strongly agree) to indicate whether or not the feature is present in the current work setting.

The sleep quality was evaluated with the Pittsburg Sleep Quality Index (PSQI) questionnaire [38,39], which consisted of 24 questions. After correction of this questionnaire, seven scores were obtained that reported the components of sleep quality: subjective quality, sleep latency, sleep duration, habitual sleep efficiency, sleep disturbances, use of hypnotic medication, and daytime dysfunction. Each component was assigned a score ranging from 0 (no difficulty) to 3 (severe difficulty), and the total score ranged from 0 to 21, which was considered the global PSQI score.

### 2.4. Ethical Aspects

The Ethics Committee of the Department of Nursing, Physiotherapy and Medicine at the University of Almeria (EFM 32/19) granted ethical approval. The study was conducted in compliance with the Declaration of Helsinki and the ethical principles of biomedical research. Data collection was designed to ensure confidentiality and anonymity, and participants gave informed consent prior to the study, with the possibility of withdrawing at any time.

### 2.5. Data Analysis

A descriptive statistical analysis was performed expressing the results as means (with standard deviation). A Kolmogorov–Smirnov test was used to assess the normality. Confidence intervals were obtained at 95% for both means and proportions considering a significant difference when *p* < 0.05. To contrast the results, Student’s t-test or the non-parametric Mann–Whitney U-test were used, as appropriate. Categorical variables were compared with the χ2 or Fisher’s exact test. On the other hand, to assess whether or not there were intergroup differences between the different short-term measurements, Student’s t-test for paired samples or Wilcoxon’s test were used in parametric and non-parametric cases, respectively. Repeated measures analysis of variance or Friedman’s test were used in parametric or non-parametric cases, respectively, to determine whether or not there were differences for each of the groups between the different long-term measurements. Data analysis was performed using IBM SPSS Statistics 25.0.

## 3. Results

The sample consisted of 380 nursing professionals, 221 of whom worked in rotating shifts (RS, 58.2%) and 159 in fixed shifts (FS, 41.8%). The sociodemographic characteristics of the study participants are shown in Table 2. The mean age of the FS was 24.8 (6.9) years, and 28.0 (6.7) years in the RS. Regarding sex, in both groups, the majority were women (FS: 93.1%; RS: 92.8%).

In reference to the diagnosis of gastrointestinal disease, 83.03% of the FS and 74.66% of the RS had no gastrointestinal disease. Likewise, most participants did not take any type of medication (FS: 69.81%; RS: 61.99%) or supplements (FS: 83.02%; RS: 76.47%) on a regular basis (Table 2).

Regarding gastrointestinal symptomatology measured with the GSRS scale, designed to assess symptoms associated with common gastrointestinal disorders, total scores were statistically higher (*p* < 0.05) in the RS group compared to the FS group. In addition, scores on the indigestion and abdominal pain subscales also increased in the RS (*p* < 0.05). Higher scores indicate worsening symptomatology. In contrast, no significant differences were found in the subscales of diarrhea, constipation, and reflux between the two groups (*p* > 0.05) (Table 3).

Regarding stool consistency measured with the Bristol scale, no statistically significant differences (*p* > 0.05) were found between the two groups in any of the three measurements performed at different times of the study (month, week, last). Most participants had normal stool consistency (Table 4).

In terms of sleep quality as measured with PSQI, only the sleep efficiency subscale, referring to the percentage of time spent sleeping compared to time spent lying in bed, was worse in the RS group compared to FS (*p* < 0.05). Scores on the overall sleep quality scale, on the subscales of charity, disturbance, duration, latency, medication use, and daytime dysfunction, showed no differences between groups (*p* > 0.05) (Table 5).

Workers in both work shifts did not show statistically different scores on the emotional exhaustion and personal accomplishment subscales of the MBI-HSS questionnaire, which assesses the presence of Burnout Syndrome. However, the scores on the depersonalization subscale were higher (*p* < 0.05) in the RS group compared to the FS group. Emotional distress was also assessed using the HADS scale in both groups of health professionals. Total scores were significantly lower (*p* < 0.05) in RS workers while no differences (*p* > 0.05) were found in the anxiety and depression subscales (Table 6).

Regarding the environment in which nursing work is performed, as measured by the PES-NWI questionnaire, all domains of this questionnaire obtained lower scores in the RS group. Statistically significant differences (*p* < 0.05) were obtained in all domains except for nurse–physician relationships (Table 7).

## 4. Discussion

Several studies suggest that shift work is related to sleep cycle disturbances and gastrointestinal and mental disorders in both healthcare and non-healthcare professionals [6,22,40]. In that context, the aim of this study was to describe the impact of RS and FS on the health of nursing professionals. In the present study, healthcare workers with RS reported more frequent gastrointestinal symptoms, less efficient sleep, emotional distress, symptoms of depersonalization, and a poorer nursing practice environment.

Gastrointestinal symptomatology appears to be a common complaint in workers subjected to high levels of stress [41,42], such as healthcare workers. In relation to the different work shifts, it has been suggested that RS may increase the occurrence of gastrointestinal problems, mainly indigestion and peptic ulcers [43]. In the present work, RS nurses reported more gastrointestinal symptoms, such as indigestion and abdominal pain. Similarly, the presence of abdominal pain and an increase in the number of gastrointestinal complaints were described more frequently in RS nurses compared to FS nurses [6,44]. In addition, the feces were characterized according to their consistency using the Bristol scale, which allowed the identification of healthy or unhealthy bowel movements. No differences in stool consistency were found between the two groups of participants. Consistent with our results, other studies also found no relationship between shift work and bowel health when measuring stool consistency and pattern, stool frequency, intestinal gas leakage, and fecal incontinence [45].

Sleep disorder due to different work shifts is characterized by an imbalance of the circadian rhythm, mainly affecting people working night and rotating shifts. This condition can cause insomnia or excessive sleepiness and, consequently, affect productivity [46,47]. In terms of sleep quality, as measured with the PSQI questionnaire, our results show similar overall scores except for the sleep efficiency subscale, which was found to be decreased in RS. This differs from other studies showing differences in PSQI scores between day and night shifts, as well as poorer subjective sleep quality, daytime dysfunction, and sleep disturbances [2,48]. At the metabolic level, cortisol release patterns may vary between early and late shifts [49]. It was reported that nurses on the evening or night shift show a lower increase in cortisol after waking and experience worse sleep quality than day shift nurses [50]. In addition, the number of days needed to adjust the nurses’ circadian patterns of cortisol secretion after the night shift was assessed and determined to be at least 4 days [20].

Although there is some controversy, it has been suggested that disturbances in circadian rhythm and, consequently, in sleep cycles are associated with gastrointestinal disorders. In relation to this, sleep deprivation in patients with GER aggravates symptoms by increasing the intensity and sensitivity to acid perfusion [51]. On the contrary, participation in shift work, especially rotating shift work, are associated with the development and prevalence of irritable bowel syndrome (IBS) and abdominal pain that is shown independent of sleep quality [44,52].

Regarding work stress or Burnout Syndrome, our results measured using the MBI-HSS questionnaire showed differences between FS and RS in the depersonalization dimension, yet they did not differ in the subscales of emotional exhaustion or burnout and personal fulfilment. These results disagree with those obtained by other researchers who showed that RS did not influence job stress measured with the Professional Quality of Life (ProQoL) Scale and the MBI-HSS [48,53]. Contrary to our results, one study showed that critical care nurses with FS experience more burnout than RS nurses. Specifically, emotional exhaustion and depersonalization scores were statistically significantly higher for FS nurses [1].

The results obtained show differences in the total scores of the HADS scale, although the anxiety and depression subscales do not show differences between participants. These results are similar to those obtained by Tahghighi et al. (2019), which shows the limited influence of RS on anxiety and depression measured with the Depression, Anxiety and Stress Scale (DASS21) [53]. However, the study by Booker et al. (2019) concluded that RS is strongly associated with the occurrence of depression and anxiety on the General Anxiety Disorder (GAD-7) scale [54].

Regarding the work environment, the findings show differences between FS and RS in all subscales of the PES-NWI questionnaire, except for the collegial nurse–physician relationships domain. Our results agree with some studies, as differences in the work environment are observed with respect to the type of work shift. However, our results show a more favorable work environment in FS, while research such as Bullich-Marín et al. (2016) and Dehring et al. (2018) show it to be more favorable in RS [55,56].

The variables examined in this study could be an important factor in RS, but it would be interesting to explain the involvement of dietary habits [57,58,59] in professionals with RS in future research. Furthermore, as previously discussed, these RS may influence sleep quality [60] and may involve circadian rhythm disturbance impacting gut microbiota composition via the HPA axis [21,61]. Thus, an alteration of the gut microbiota may generate a disturbance of complex cognitive functions, such as thinking and decision-making [62], and, consequently, have a positive or negative effect on the quality of the medical attention provided.

Taking into account all of the above, future research should execute objective evaluations to assess the physical and psychological status of healthcare professionals. Another important point is the exploration of the role of gut microbiota in the health of professionals and the impact of shift work through the exploration of 16S ribosomal RNA gene sequences, as recent studies have shown how the gut microbiota influences the circadian rhythm [21], sleep quality [61], stress [21], and gastrointestinal disease [63].

## 5. Conclusions

The present study suggests that the type of shift work may influence nurses physically and emotionally. Gastrointestinal symptoms, impaired sleep efficiency, depersonalization, and an inadequate work environment can often appear in nurses on rotating shifts. It is clear that nursing is an arduous and exhausting profession, often associated with psychological distress. These findings will support the implementation of prevention and intervention programs at the individual, group, and organizational levels to succeed in reaching gastrointestinal homeostasis.

## Figures and Tables

**Table 1:** Criteria for inclusion and exclusion of participants.

Inclusion Criteria	Exclusion Criteria
Active nurses with more than 1 year of seniority in the service	Gastrointestinal pathology
Fixed group: Fixed mornings, afternoons, or evenings	Severe mental illness
Rotating group: Rotating shifts	Professionals with reduced working hours
Full-time professionals	Working in more than two organizations
Voluntary participation	Participation in another study interfering with the results

**Table 2:** Sociodemographic characteristics of participants according to work shift.

	Total (*n* = 380)	Fixed Shift (*n* = 159)	Rotating Shift (*n* = 221)	*p*
Age (Mean (SD))	24.8 (6.9)	24.8 (6.9)	28.0 (6.7)	<0.0001
Sex (%)				
Man	7.1 (*n* = 27)	6.9 (*n* = 11)	7.2 (*n* = 16)	0.90
Woman	92.9 (*n* = 353)	93.1 (*n* = 148)	92.8 (*n* = 205)	
Marital status (%)				
Single	40.0 (*n* = 152)	50.94 (*n* = 81)	32.13 (*n* = 71)	0.001
With partner	12.63 (*n* = 48)	8.81 (*n* = 14)	15.38 (*n* = 34)	
Married	47.11 (*n* = 179)	40.25 (*n* = 64)	52.04 (*n* = 115)	
Divorced	0.26 (*n* = 1)	0.0 (*n* = 0)	0.45 (*n* = 1)	
No. of children				
Mean (SD)	0.20 (0.6)	0.2 (0.6)	0.2 (0.6)	0.07
Gastrointestinal disease (%)				
Yes	21.84 (*n* = 83)	16.98 (*n* = 27)	25.34 (*n* = 56)	0.05
No	72.5 (*n* = 182)	83.02 (*n* = 132)	74.66 (*n* = 165)	
Consumption of medication (%)				
Yes	34.74 (*n* = 132)	30.19 (*n* = 48)	38.01 (*n* = 84)	0.11
No	65.26 (*n* = 248)	69.81 (*n* = 111)	61.99 (*n* = 137)	
Consumption of supplements (%)				
Yes	20.79 (*n* = 79)	16.98 (*n* = 27)	23.53 (*n* = 52)	0.12
No	79.21 (*n* = 301)	83.02 (*n* = 132)	76.47 (*n* = 169)	

SD: Standard deviation.

**Table 3:** GSRS scores.

Dimension (Score Range)	Fixed Shift (Mean (SD))	Rotating Shift (Mean (SD))	*p*
Indigestion (4–28)	11.9 (5.8)	13.5 (6.6)	0.03
Abdominal pain (3–21)	7.6 (2.9)	8.6 (3.6)	0.01
Diarrhea (3–21)	7.0 (4.6)	8.0 (5.0)	0.05
Constipation (3–21)	7.5 (4.8)	8.0 (5.0)	0.26
Reflux (2–14)	3.5 (2.4)	4.1 (3.0)	0.09
GSRS global score (15–105)	37.5 (15.5)	42.2 (18.7)	0.02

GSRS: Gastrointestinal Symptom Rating Scale; SD: standard deviation.

**Table 4:** Bristol scores.

		Fixed Shift % (*n*)	Rotating Shift % (*n*)	*p*
Last month deposition	Constipation	13.84 (*n* = 22)	11.76 (*n* = 26)	0.45
	Normal	69.81 (*n* = 111)	66.97 (*n* = 148)	
	Diarrhea	16.35 (*n* = 26)	21.27 (*n* = 47)	
Last week deposition	Constipation	15.72 (*n* = 25)	14.93 (*n* = 33)	0.26
	Normal	63.52 (*n* = 101)	57.01 (*n* = 126)	
	Diarrhea	20.75 (*n* = 33)	28.05 (*n* = 62)	
Last deposition	Constipation	10.69 (*n* = 17)	15.38 (*n* = 34)	0.26
	Normal	63.52 (*n* = 101)	56.11 (*n* = 124)	
	Diarrhea	25.79 (*n* = 41)	28.51 (*n* = 63)	

**Table 5:** PSQI scores.

Dimension (Score Range)	Fixed Shift (Mean (SD))	Rotating Shift (Mean (SD))	*p*
Sleep quality (0–3)	1.2 (1.4)	1.4 (1.4)	0.1
Sleep efficiency (0–3)	0.6 (0.8)	0.9 (1.1)	0.01
Sleep disturbance (0–3)	1.3 (0.5)	1.3 (0.5)	0.29
Sleep duration (0–3)	1.5 (0.8)	1.5 (0.9)	0.89
Sleep onset latency (0–3)	1.7 (0.9)	1.7 (1.0)	0.34
Hypnotic drugs (0–3)	0.3 (0.8)	0.3 (0.6)	0.95
Daytime dysfunction (0–3)	1.6 (0.8)	1.6 (0.8)	0.99
PSQI global score (0–21)	9.2 (4.2)	10.0 (4.6)	0.1

PSQI: Pittsburgh Sleep Quality Index; SD: standard deviation.

**Table 6:** MBI-HSS and HADS scores.

**MBI-HSS questionnaire**			
**Dimension (Score range)**	**Fixed shift (Mean (SD))**	**Rotating shift (Mean (SD))**	***p***
Emotional exhaustion (0–54)	19.63 (10.60)	22.64 (11.35)	0.07
Depersonalization (0–30)	5.50 (4.88)	6.86 (5.86)	0.01
Personal accomplishment (0–48)	32.62 (8.07)	32.62 (8.07)	0.89
**HADS questionnaire**			
**Dimension (Score range)**	**Fixed shift (Mean (SD))**	**Rotating shift (Mean (SD))**	***p***
Anxiety (0–21)	11.7 (2.6)	11.1 (2.8)	0.08
Depression (0–21)	7.9 (2.8)	7.5 (2.9)	0.11
HADS global score (0–42)	19.6 (3.5)	18.6 (3.7)	0.02

MBI-HSS: Maslach Burnout Inventory-Human Services Survey; HADS: Hospital Anxiety and Depression Scale; SD: standard deviation.

**Table 7:** PES-NWI scores.

Dimension (Score Range)	Fixed Shift (Mean (SD))	Rotating Shift (Mean (SD))	*p*
Nurse participation in hospital affairs (8–32)	21.0 (5.3)	18.7 (5.9)	0.0001
Nursing foundations for quality of care (9–36)	26.8 (5.9)	24.4 (6.4)	0.0002
Nurse manager ability leadership and support of nurses (4–16)	13.8 (3.7)	12.8 (4.4)	0.04
Staffing and resource adequacy (4–16)	7.9 (3.0)	7.1 (3.1)	0.04
Collegial nurse–physician relationships (7–28)	8.2 (2.3)	7.7 (2.6)	0.10

PES-NWI: Practice Environment Scale from Revised Nurse Work Index; SD: standard deviation.

## Data Availability

The data that support the findings of this study are available on request from the corresponding author.

## References

[1] Shahriari M., Shamali M., Yazdannik A. (2014). The Relationship between Fixed and Rotating Shifts with Job Burnout in Nurses Working in Critical Care Areas. Iran. J. Nurs. Midwifery Res..

[2] Ferri P., Guadi M., Marcheselli L., Balduzzi S., Magnani D., Di Lorenzo R. (2016). The Impact of Shift Work on the Psychological and Physical Health of Nurses in a General Hospital: A Comparison between Rotating Night Shifts and Day Shifts. Risk Manag. Healthc. Policy.

[3] Selvi Y., Özdemir P., Özdemir O., Aydin A., Besiroglu L. (2010). Influence of Night Shift Work on Psychologic State and Quality of Life in Health Workers. Düşünen Adam.

[4] Šimunić A., Gregov L. (2012). Conflict between Work and Family Roles and Satisfaction among Nurses in Different Shift Systems in Croatia: A Questionnaire Survey. Arh. Hig. Rada Toksikol..

[5] Strzemecka J., Bojar I., Strzemecka E., Owoc A. (2014). Dietary Habits among Persons Hired on Shift Work. Ann. Agric. Environ. Med..

[6] Saberi H.R., Moravveji A.R. (2010). Gastrointestinal Complaints in Shift-Working and Day-Working Nurses in Iran. J. Circadian Thythms.

[7] Li Y.M., Du J., Zhang H., Yu C.H. (2008). Epidemiological Investigation in Outpatients with Symptomatic Gastroesophageal Reflux from the Department of Medicine in Zhejiang Province, East China. J. Gastroenterol. Hepatol..

[8] Konturek P.C., Brzozowski T., Konturek S.J. (2011). Gut Clock: Implication of Circadian Rhythms in the Gastrointestinal Tract. J. Physiol. Pharmacol..

[9] Voigt R.M., Forsyth C.B., Keshavarzian A. (2019). Circadian Rhythms: A Regulator of Gastrointestinal Health and Dysfunction. Expert Rev. Gastroenterol. Hepatol..

[10] James S.M., Honn K.A., Gaddameedhi S., Van Dongen H.P.A. (2017). Shift Work: Disrupted Circadian Rhythms and Sleep—Implications for Health and Well-Being. Curr. Sleep Med. Rep..

[11] Stepanski E.J. (2002). The Effect of Sleep Fragmentation on Daytime Function. Sleep.

[12] Mizuno K., Matsumoto A., Aiba T., Abe T., Ohshima H., Takahashi M., Inoue Y. (2016). Sleep Patterns among Shift-Working Flight Controllers of the International Space Station: An Observational Study on the JAXA Flight Control Team. J. Physiol. Anthropol..

[13] Zhang L., Sun D.M., Li C.B., Tao M.F. (2016). Influencing Factors for Sleep Quality among Shift-Working Nurses: A Cross-Sectional Study in China Using 3-Factor Pittsburgh Sleep Quality Index. Asian Nurs. Res..

[14] Cheng P., Tallent G., Bender T.J., Tran K.M., Drake C.L. (2017). Shift Work and Cognitive Flexibility: Decomposing Task Performance. J. Biol. Rhythms.

[15] Deguchi Y., Iwasaki S., Ishimoto H., Ogawa K., Fukuda Y., Nitta T., Mitake T., Nogi Y., Inoue K. (2017). Relationships between Temperaments, Occupational Stress, and Insomnia among Japanese Workers. PLoS ONE.

[16] Seong J., Son S., Min A. (2022). Effect of Sleep on Alertness at Work among Fixed Night Shift Nurses: A Prospective Observational Study. J. Adv. Nurs..

[17] Chen G.X., Fang Y., Guo F., Hanowski R.J. (2016). The Influence of Daily Sleep Patterns of Commercial Truck Drivers on Driving Performance. Accid. Anal. Prev..

[18] Harris A., Waage S., Ursin H., Hansen Å.M., Bjorvatn B., Eriksen H.R. (2010). Cortisol, Reaction Time Test and Health among Offshore Shift Workers. Psychoneuroendocrinology.

[19] Ritonja J., Aronson K.J., Day A.G., Korsiak J., Tranmer J. (2018). Investigating Cortisol Production and Pattern as Mediators in the Relationship between Shift Work and Cardiometabolic Risk. Can. J. Cardiol..

[20] Niu S.F., Chung M.H., Chu H., Tsai J.C., Lin C.C., Liao Y.M., Ou K.L., O´Brien A.P., Chou K.R. (2015). Differences in Cortisol Profiles and Circadian Adjustment Time between Nurses Working Night Shifts and Regular Day Shifts: A Prospective Longitudinal Study. Int. J. Nurs. Stud..

[21] Reynolds A.C., Paterson J.L., Ferguson S.A., Stanley D., Wright K.P., Dawson D. (2017). The Shift Work and Health Research Agenda: Considering Changes in Gut Microbiota as a Pathway Linking Shift Work, Sleep Loss and Circadian Misalignment, and Metabolic Disease. Sleep Med. Rev..

[22] Maslach C., Leiter M.P. (2016). Understanding the Burnout Experience: Recent Research and Its Implications for Psychiatry. World Psychiatry.

[23] De Hert S. (2020). Burnout in Healthcare Workers: Prevalence, Impact and Preventative Strategies. Local Reg. Anesth..

[24] Shah M.K., Gandrakota N., Cimiotti J.P., Ghose N., Moore M., Ali M.K. (2021). Prevalence of and Factors Associated with Nurse Burnout in the US. JAMA Netw. Open.

[25] Vlasak T., Dujlovic T., Barth A. (2022). Neurocognitive Impairment in Night and Shift Workers: A Meta-Analysis of Observational Studies. Occup. Environ. Med..

[26] von Elm E., Altman D.G., Egger M., Pocock S.J., Gøtzsche P.C., Vandenbroucke J.P. (2008). The Strengthening the Reporting of Observational Studies in Epidemiology (STROBE) Statement: Guidelines for Reporting Observational Studies. J. Clin. Epidemiol..

[27] Instituto Nacional de Estadística (2019). No de Enfermeros Por Comunidades, Ciudades Autónomas y Provincias de Colegiación, Edad y Sexo.

[28] Kulich K.R., Pique J.M., Vegazo O., Jimenez J., Zapardiel J., Carlsson J., Wiklund I. (2005). Psychometric Validation of Translation to Spanish of the Gastrointestinal Symptoms Rating Scale (GSRS) and Quality of Life in Reflux and Dyspepsia (QOLRAD) in Patients with Gastroesophageal Reflux Disease. Rev. Clin. Esp..

[29] Parés D., Comas M., Dorcaratto D., Araujo M.I., Vial M., Bohle B., Pera M., Grande L. (2009). Adaptation and Validation of the Bristol Scale Stool Form Translated into the Spanish Language among Health Professionals and Patients. Rev. Esp. Enferm. Dig..

[30] Spiller R.C. (2004). Irritable Bowel Syndrome. Br. Med. Bull..

[31] Martinez A.P., Regina De Azevedo G. (2012). The Bristol Stool Form Scale: Its Translation to Portuguese, Cultural Adaptation and Validation. Rev. Lat. Am. Enferm..

[32] Maslach C., Jackson S.E., Leiter M.P., Zalaquett C.P., Wood R.J. (1997). Maslach Burnout Inventory Manual. Evaluating Stress: A Book of Resources.

[33] Gil-Monte P., Viotti S., Converso D. (2017). Psychometric Properties of the «Spanish Burnout Inventory»(SBI) in a Sample of Italian Health Professionals: A Gender Perspective. Liberabit.

[34] López-Roig S., Terol M.C., Pastor M.A., Neipp M.C., Massutí B. (2000). Ansiedad y Depresión. Validación de La Escala HAD En Pacientes Oncológicos. Rev. Psicol. Salud.

[35] Zigmond A.S., Snaith R.P. (1983). The Hospital Anxiety and Depression Scale. Acta Psychiatr. Scand..

[36] Lake E.T. (2002). Development of the Practice Environment Scale of the Nursing Work Index. Res. Nurs. Health.

[37] Fuentelsaz-Gallego C., Moreno-Casbas M.T., González-María E. (2013). Validation of the Spanish Version of the Questionnaire Practice Environment Scale of the Nursing Work Index. Int. J. Nurs. Stud..

[38] Buysse D.J., Reynolds C.F., Monk T.H., Berman S.R., Kupfer D.J. (1989). The Pittsburgh Sleep Quality Index: A New Instrument for Psychiatric Practice and Research. Psychiatry Res..

[39] Macías J.A., Royuela A. (1996). La Versión Española Del Índice de Calidad de Sueño de Pittsburgh. Inf. Psiquiátricas.

[40] Patterson P.D., Ghen J.D., Antoon S.F., Martin-Gill C., Guyette F.X., Weiss P.M., Turner R.L., Buysse D.J. (2019). Does Evidence Support “Banking/Extending Sleep” by Shift Workers to Mitigate Fatigue, and/or to Improve Health, Safety, or Performance? A Systematic Review. Sleep Health.

[41] Nam Y., Kwon S.C., Lee Y.J., Jang E.C., Ahn S.H. (2018). Relationship between Job Stress and Functional Dyspepsia in Display Manufacturing Sector Workers: A Cross-Sectional Study. Ann. Occup. Environ. Med..

[42] Huerta-Franco M.-R., Vargas-Luna M., Tienda P., Delgadillo-Holtfort I., Balleza-Ordaz M., Flores-Hernandez C. (2013). Effects of Occupational Stress on the Gastrointestinal Tract. World J. Gastrointest. Pathophysiol..

[43] Chang W.P., Peng Y.X. (2021). Differences between Fixed Day Shift Workers and Rotating Shift Workers in Gastrointestinal Problems: A Systematic Review and Meta-Analysis. Ind. Health.

[44] Nojkov B., Rubenstein J.H., Chey W.D., Hoogerwerf W.A. (2010). The Impact of Rotating Shift Work on the Prevalence of Irritable Bowel Syndrome in Nurses. Am. J. Gastroenterol..

[45] Storz M.A., Lombardo M., Rizzo G., Müller A., Lederer A.K. (2022). Bowel Sealth in U.S. Shift Workers: Insights from a Cross-Sectional Study (NHANES). Int. J. Environ. Res. Public Health.

[46] Wickwire E.M., Geiger-Brown J., Scharf S.M., Drake C.L. (2017). Shift Work and Shift Work Sleep Disorder: Clinical and Organizational Perspectives. Chest.

[47] Park E., Lee H.Y., Park C.S.Y. (2018). Association between Sleep Quality and Nurse Productivity among Korean Clinical Nurses. J. Nurs. Manag..

[48] Gomez-Garcia T., Ruzafa-Martinez M., Fuentelsaz-Gallego C., Madrid J.A., Rol M.A., Martinez-Madrid M.J., Moreno-Casbas T. (2016). Nurses’ Sleep Quality, Work Environment and Quality of Care in the Spanish National Health System: Observational Study among Different Shifts. BMJ Open.

[49] Bostock S., Steptoe A. (2013). Influences of Early Shift Work on the Diurnal Cortisol Rhythm, Mood and Sleep: Within-Subject Variation in Male Airline Pilots. Psychoneuroendocrinology.

[50] Chang W.P. (2018). Influence of Shift Type on Sleep Quality of Female Nurses Working Monthly Rotating Shifts with Cortisol Awakening Response as Mediating Variable. Chronobiol. Int..

[51] Schey R., Dickman R., Parthasarathy S., Quan S.F., Wendel C., Merchant J., Powers J., Han B., van Handel D., Fass R. (2007). Sleep Deprivation Is Hyperalgesic in Patients With Gastroesophageal Reflux Disease. Gastroenterology.

[52] Wells M.M., Roth L., Chande N. (2012). Sleep Disruption Secondary to Overnight Call Shifts Is Associated with Irritable Bowel Syndrome in Residents: A Cross-Sectional Study. Am. J. Gastroenterol..

[53] Tahghighi M., Brown J.A., Breen L.J., Kane R., Hegney D., Rees C.S. (2019). A Comparison of Nurse Shift Workers’ and Non-Shift Workers’ Psychological Functioning and Resilience. J. Adv. Nurs..

[54] Booker L.A., Sletten T.L., Alvaro P.K., Barnes M., Collins A., Chai-Coetzer C.L., Naqvi A., McMahon M., Lockley S.W., Rajaratnam S.M.W. (2020). Exploring the Associations between Shift Work Disorder, Depression, Anxiety and Sick Leave Taken amongst Nurses. J. Sleep Res..

[55] Bullich-Marín I., Miralles Basseda R., Torres Egea P., Planas-Campmany C., Juvé-Udina M.E. (2016). Evaluation of the Nurse Working Environment in Health and Social Care Intermediate Care Units in Catalonia. Rev. Esp. Geriatr. Gerontol..

[56] Dehring T., Von Treuer K., Redley B. (2018). The Impact of Shift Work and Organisational Climate on Nurse Health: A Cross-Sectional Study. BMC Health Serv. Res..

[57] Pei-Chen L., Chung-Hey C., Shung-Mei P., Yao-Mei C., Chih-Hong P., Hsin-Chia H., Ming-Tsang W. (2015). The Association between Rotating Shift Work and Increased Occupational Stress in Nurses. J. Occup. Health.

[58] von Treuer K., Fuller-Tyszkiewicz M., Little G. (2014). The Impact of Shift Work and Organizational Work Climate on Health Outcomes in Nurses. J. Occup. Health Psychol..

[59] Dehghan H., Azmoon H., Souri S., Akbari J. (2014). The Effects of State Anxiety and Thermal Comfort on Sleep Quality and Eye Fatigue in Shift Work Nurses. J. Educ. Health Promot..

[60] Beebe D., Chang J.J., Kress K., Mattfeldt-Beman M. (2017). Diet Quality and Sleep Quality among Day and Night Shift Nurses. J. Nurs. Manag..

[61] Kaczmarek J.L., Thompson S.V., Holscher H.D. (2017). Complex Interactions of Circadian Rhythms, Eating Behaviors, and the Gastrointestinal Microbiota and Their Potential Impact on Health. Nutr. Rev..

[62] Ma H., Li Y., Liang H., Chen S., Pan S., Chang L., Li S., Zhang Y., Liu X., Xu Y. (2019). Sleep Deprivation and a Non-24-h Working Schedule Lead to Extensive Alterations in Physiology and Behavior. FASEB J..

[63] Nobs S.P., Tuganbaev T., Elinav E. (2019). Microbiome Diurnal Rhythmicity and Its Impact on Host Physiology and Disease Risk. EMBO Rep..

